# Patients undergoing colorectal surgery at a Veterans Affairs Hospital do not experience racial disparity in length of stay either before or after implementing an enhanced recovery pathway

**DOI:** 10.1186/s12893-022-01647-3

**Published:** 2022-05-21

**Authors:** C. Rentas, S. Baker, L. Goss, J. Richman, S. J. Knight, C. Key, M. Morris

**Affiliations:** 1 Department of Surgery, University of Alabama at Birmingham School of Medicine, 1808 7th Avenue South #503, Birmingham, AL 35233 USA; 2 Birmingham VA Medical Center, 700 19th Street South, Birmingham, AL 35233 USA; 3 VA Salt Lake City Health Care System, University of Utah, 500 Foothill Dr, Salt Lake City, UT 84148 USA

**Keywords:** Enhanced Recovery Pathways, Veterans Affairs, Disparities, Race

## Abstract

**Background:**

Enhanced Recovery Pathways (ERP) have been shown to reduce racial disparities following surgery. The objective of this study is to determine whether ERP implementation mitigates racial disparities at a Veterans Affairs Hospital.

**Methods:**

A retrospective cohort study was conducted using data obtained from the Veterans Affairs Surgical Quality Improvement Program. All patients undergoing elective colorectal surgery following ERP implementation were included. Current procedural terminology (CPT) codes were used to identify patients who underwent similar procedures prior to ERP implementation.

**Results:**

Our study included 417 patients (314 pre-ERP vs. 103 ERP), 97.1% of which were male, with an average age of 62.32 (interquartile range (IQR): 25–90). ERP patients overall had a significantly shorter post-operative length of stay (pLOS) vs. pre-ERP patients (median 4 days (IQR: 3–6.5) vs. 6 days (IQR: 4–9) days (p < 0.001)). Within the pre-ERP group, median pLOS for both races was 6 days (IQR: 4–6; p < 0.976) and both groups experienced a decrease in median pLOS (4 vs. 6 days; p < 0.009 and p < 0.001) following ERP implementation.

**Conclusions:**

Racial disparities did not exist in patients undergoing elective surgery at a single VA Medical Center. Implementation of an ERP significantly reduced pLOS for black and white patients.

**Supplementary Information:**

The online version contains supplementary material available at 10.1186/s12893-022-01647-3.

## Background

In the United States, racial inequalities exist in the onset, course, outcome of illness, and in post-operative outcomes across surgical specialties [1–4]. Studies have shown that black patients experience longer post-operative lengths of stay (pLOS), more complications, and higher rates of mortality when compared with white patients [2, 5–8]. It remains unclear why these disparities exist and increased federal funding has been approved to understand these disparities as well as develop strategies to mitigate them [9].

One such strategy relies on the implementation of standardized protocols in the perioperative setting. Enhanced Recovery Pathways (ERP) are highly effective multimodal, evidence-based approaches intended to optimize surgical outcomes by reducing variations in patient care. For example, ERP implementation has consistently been shown to reduce post-operative complication rates without increasing cost or readmission rates [6, 10–13]. A growing body of literature supports the role of ERP in reducing racial inequality in surgical care [14, 15]. Notably, ERP implementation was recently shown to reduce racial disparities in post-operative length of stay at a large academic medical center [16, 17]. Although the mechanism by which ERP reduces racial inequality is unknown, some hypotheses include: improved patient education, mitigation of implicit or explicit bias, and better adherence to evidence-based care practices, though more studied are needed to understand the exact mechanism [14].

The US Department of Veterans Affairs (VA) healthcare system provides surgical care to a racially diverse population without the financial barriers that exist in other healthcare settings [18]. It is unknown if racial disparities exist following elective colorectal surgery in the VA healthcare system. In addition, little is known about ERP implementation within the Veterans Affairs Hospital System and its effect on post-operative outcomes. We hypothesized that racial disparities in pLOS exist among patients at a single Veterans Affairs Hospital and implementation of an ERP protocol would mitigate these disparities.

## Material and methods

In January 2016, an ERP protocol was implemented at the Birmingham VA Medical Center. All patients undergoing elective colorectal procedures were enrolled in the ERP with no inclusion or exclusion criteria applied. A retrospective cohort study was conducted to assess differences in post-operative outcomes between black and white patients before and after the ERP protocol was implemented at our institution. All patients that underwent an operation using the ERP protocol between January 2016 and March 2018 were identified as ERP patients. Current procedural terminology (CPT) Codes identified pre-ERP patients that underwent similar operations from January 2010 to January 2016.

Demographic and procedural information was retrospectively collected through the national, VA-wide database Veterans Affairs Surgical Quality Improvement Program (VASQIP) [19]. Patients who underwent emergent surgery or who had an in-hospital mortality were excluded from the study. The primary outcome assessed was post-operative length of stay (pLOS) stratified by the two racial groups (black vs. white). Patients reported their race on hospital intake forms and chart-abstraction was then used to assign patient race for the purposes of this study. Non-black, non-white and patients of unknown race were excluded from our analysis due to low numbers (15 patients). Secondary outcomes include: 30-day VASQIP-assessed post-operative complications, readmission rates, and ERP protocol adherence [19]. Included cases that were not assessed by VASQIP were manually chart abstracted.

Procedures were classified into four categories, as listed in Table 1. Abdominal Perineal Resection (APR), Low Anterior Resection (LAR), Proctectomy, and Altemeier (Perineal Rectosigmoidectomy) were all classified as “Rectal” procedures. Hemi-colectomy and Sigmoidectomy procedures were classified as “Partial Colectomy”. Ileostomy procedures were classified as “Small Bowel” operations, and TAC/TPC refers to “Total Abdominal Colectomy/Total Proctocolectomy”.

**Table 1:** Patient and procedure characteristics

	Overall (N = 417)	Pre-ERP (n = 314)	ERP (n = 103)	*p*
Patient level				
Age, mean (SD)	62.32 (9.7)	62.11 (9.82)	62.96 (9.34)	0.440
BMI, mean (SD)	27.92 (5.5)	27.69 (5.34)	28.61 (5.83)	0.140
Sex				0.080
Female	12 (2.9)	6 (1.9)	6 (5.8)	
Male	404 (97.1)	308 (98.1)	96 (93.2)	
Smoker	175 (42.0)	140 (44.6)	35 (34.0)	0.076
Hypertension		194 (64.4)	66 (66.7)	0.767
Diabetes	95 (22.8)	71 (22.6)	24 (23.3)	0.524
ASA Class				0.025
1	2 (0.2)	2 (0.6)	0 (0.0)	
2	28 (6.7)	15 (4.8)	13 (12.6)	
3	360 (86.3)	274 (87.2)	86 (83.5)	
4	27 (6.5)	23 (7.3)	4 (3.9)	
Operative level				
Procedure				0.001
Partial colectomy	210 (52.1)	141 (46.5)	67 (69.8)	
Rectal	40 (9.9)	25 (8.3)	14 (14.6)	
Small Bowel	148 (36.7)	134 (44.2)	14 (14.6)	
TAC/TPC	5 (1.2)	3 (1.0)	1 (1.0)	

All data represented as n (column %) unless otherwise specified

ASA Class, American Society of Anesthesiologist Class; BMI, body mass index; TAC/TPC, total abdominal colectomy/total proctocolectomy

“Overall” includes non-black, non-white patients and those whose race is unknown

Prior to implementation of the ERP in 2016, perioperative management at the Birmingham VA Medical Center was not standardized. The ERP protocol was implemented based on the ERAS Society Guidelines for Colorectal Surgery and mirrors the ERP instituted in our academic affiliate hospital, the University of Alabama at Birmingham as published by Wahl et al. [16]. Patients undergoing elective colorectal procedures were identified in clinic and subsequently educated on ERP. Pre-operatively, patients were prescribed oral antibiotics and mechanical bowel prep when indicated and were instructed to consume a carbohydrate-rich drink the morning of surgery. Multi-modal pain management was administered via oral acetaminophen, celecoxib, and gabapentin in addition to intrathecal spinal analgesia. Patients were assessed for risk of post-operative nausea and vomiting and were treated prophylactically if indicated. Post-operative pain management relied minimally on opioids.

Pre-ERP and ERP patients were compared by patient and procedure-specific characteristics, and by primary and secondary outcomes. T-tests and Wilcoxon tests were used to compare continuous variables, and Chi-square tests were used for categorical variables. For comparisons of categorical variables where the smallest cell-count was ≤ 5, p-values were derived using 2000 Monte Carlo simulations [20]. Pre-ERP and ERP patients were then stratified by race and compared in an identical method. A Fisher’s exact test was used to calculate the power difference in rates between pre-ERP and ERP patients. A two-sided test was used to calculate the power difference in means.

There are 17 core-elements of the perioperative pathway as defined by the ERAS Society (Table 2) including patient education, minimally invasive surgical techniques, and guidelines for post-operative mobilization [6]. ERP adherence was calculated as the number of components fulfilled divided by the total possible 17 components assessed based on the ERAS guideline recommendations [21]. Missing items (whether not done or undocumented) were counted as not adherent. Patients who received or adhered to 12 out of 17 components (70%), based on previously published literature [16] were considered ERP adherent. All analyses were performed using R Core Team (2019), with an alpha level of 0.05 considered statistically significant.

**Table 2:** Components of ERP adherence

Preoperative	
Education	
Bowel Prep	
Fasting and carbohydrate treatment	
Preanesthestic medication	
Deep venous thrombosis (DVT) prophylaxis	
Antimicrobial prophylaxis	
Intraoperative	
Postoperative nausea and vomiting prophylaxis (PONV)	
Standardized anesthesia	
Minimally invasive approach	
Nasogastric intubation	
Hypothermia prevention	
Perioperative fluid management	
Postoperative	
Urinary drainage	
Prevention of postoperative ileus	
Postoperative analgesia	
Nutrition	
Early mobilization	

This table outlines the 17 components we considered in assessing ERP adherence. These components are based on the published ERAS guideline recommendations [20]

## Results

Of 417 patients included (314 pre-ERP vs. 103 ERP), 35.0% were black. The average age of the cohort was 62.3 (SD 9.7) and 97.1% were male. No differences in demographic variables including sex, BMI, and age were observed between pre-ERP and ERP patients as shown in Table 1. Additionally, pre-ERP and ERP groups were similar in race, history of diabetes, and smoking status. The pre-ERP and ERP groups differed with regards to ASA class (p < 0.025) and procedure type (p < 0.001).

When stratified by race, black and white patients were similar in terms of sex, BMI, smoking status, rates of hypertension and diabetes across the pre-ERP and ERP groups. In the pre-ERP group, white patients were older than black patients (63.1 vs. 60.3 years (p = 0.02)) (Table 3). White patients in the pre-ERP group were similar to white patients in the ERP group in all compared patient characteristics, but black patients had a slightly higher ASA class in the pre-ERP group when compared to the ERP group (Table 3).

**Table 3:** Race-stratified patient and procedure characteristics

	Pre-ERP				ERP			
	Overall (N = 314)	Black (n = 110)	White (n = 193)	p	Overall (N = 103)	Black (n = 36)	White (n = 63)	p
Age, mean (SD)	62.11 (9.82)	60.27 (9.25)	63.09 (10.2)	0.017	62.96 (9.34)	61.83 (8.72)	64.14 (9.45)	0.232
BMI, mean (SD)	27.69 (5.34)	27.05 (5.08)	28.09 (5.49)	0.104	28.61 (5.83)	29.53 (7.11)	27.95 (4.95)	0.196
Sex				0.689				0.361
Female	6 (1.9)	3 (2.7)	3 (1.6)		6 (5.8)	3 (8.3)	2 (3.2)	
Male	308 (98.1)	107 (97.3)	190 (98.4)		96 (93.2)	33 (91.7)	60 (95.2)	
Smoker	140 (44.6)	51 (46.4)	83 (43.0)	0.656	35 (34.0)	11 (30.6)	23 (36.5)	0.670
Hypertension	194 (64.4)	78 (40.0)	117 (60.0)	0.094	66 (66.7)	26 (39.4)	40 (60.6)	0.528
Diabetes	71 (22.6)	27 (24.5)	42 (21.8)	0.847	24 (23.3)	11 (30.6)	12 (19.0)	0.430
ASA class				0.242				0.326
1	2 (0.6)	1 (0.9)	1 (0.5)		0 (0.0)	0 (0.0)	0 (0.0)	
2	15 (4.8)	2 (1.8)	13 (6.7)		13 (12.6)	5 (13.9)	6 (9.5)	
3	274 (87.2)	101 (91.8)	165 (85.5)		86 (83.5)	31 (86.1)	53 (84.1)	
4	23 (7.3)	6 (5.5)	14 (7.3)		4 (3.9)	0 (0.0)	4 (6.3)	
Operative level								
Procedure				0.764				0.895
Partial colectomy	141 (46.5)	51 (36.2)	90 (63.8)		67 (69.8)	25 (37.3)	42 (62.7)	
Rectal	25 (8.3)	8 (32.0)	17 (68.0)		14 (14.6)	5 (35.7)	9 (64.3)	
Small Bowel	134 (44.2)	49 (36.6)	85 (63.4)		14 (14.6)	4 (28.6)	10 (71.4)	
TAC/TPC	3 (1.0)	2 (66.7)	1 (33.3)		1 (1.0)	0 (0.0)	1 (100.0)	

All data represented as n (column %) unless otherwise specified

ASA Class, American Society of Anesthesiologist Class; BMI, body mass index; TAC/TPC, total abdominal colectomy/total proctocolectomy

“Overall” includes non-black, non-white patients and those whose race is unknown

### Primary outcome

Overall, ERP patients had a significantly shorter pLOS (median 4 [interquartile range (IQR) 3–6.5] vs 6 (IQR 4–9) days; p < 0.001) when compared with pre-ERP patients. Within the pre-ERP group, median pLOS for both black and white patients was 6 days (IQR: 4–6; p = 0.98). Both groups experienced a decrease in median pLOS (4 vs 6, p < 0.009 and p < 0.001, respectively) following ERP implementation. Additionally, no significant difference in pLOS between black and white patients following ERP implementation was observed (p = 0.61) (Table 4).

**Table 4:** Post-operative length of stay pre and post-ERP

	Pre-ERP				ERP				
	Overall (N = 314)	Black (n = 110)	White (n = 193)	p	Overall (N = 103)	Black (n = 36)	White (n = 63)	p	Overall p
Median, IQR	6 (4.0–9.0)	6 (4.0–9.0)	6 (4.0–9.0)	0.976	4 (3.0–6.5)	4 (3.0–8.5)	4 (2.5–6.0)	0.6122	0.001
Mean, SD	8.4 (8.4)	8.5 (8.2)	8.0 (7.3)	0.607	5.8 (5.3)	6.7 (6.2)	5.1 (4.4)	0.1382	0.003

All data represented as n (column %) unless otherwise specified

“Overall” includes non-black, non-white patients and those whose race is unknown

IQR, interquartile range; SD, standard deviation

### Secondary outcomes

Following implementation of ERP, patients experienced reduced pLOS without increased 30-day readmission (16.5% vs 11.8%, p = 0.28). Additionally, there was no significant increase in VASQIP assessed post-operative complications following ERP implementation (22% vs. 25.2%, p = 0.58). Within the pre-ERP cohort, white patients experienced higher rates of 30-day readmission than black patients (15.0% vs. 6.4%, p = 0.04), but following ERP implementation that difference was mitigated (13.9% vs. 19.0%, p = 0.58). There were no significant differences in any 30-day complications when patients were stratified by race within each cohort (Table 5). Overall, racial disparities were not observed following the ERP intervention with black and white patients being similar in all outcomes assessed (Table 5).

**Table 5:** 30-day secondary outcome assessment

	Pre-ERP				ERP				
	Overall (N = 314)	Black (n = 110)	White (n = 193)	p	Overall (N = 103)	Black (n = 36)	White (n = 63)	p	Overall p
Readmission	37 (11.8)	7 (6.4)	29 (15.0)	0.040	17 (16.5)	5 (13.9)	12 (19.0)	0.580	0.280
Any complication	69 (22.0)	20 (18.2)	47 (24.4)	0.271	26 (25.2)	11 (30.6)	14 (22.2)	0.443	0.580
1 complication	49 (15.6)	13 (11.8)	35 (18.1)		14 (13.6)	5 (13.9)	9 (14.3)		
2 complication	13 (4.1)	5 (4.5)	7 (3.6)		6 (5.8)	2 (5.6)	3 (4.8)		
3 complication	4 (1.3)	0 (0.0)	4 (2.1)		5 (4.9)	3 (8.3)	2 (3.2)		
4 complication	2 (0.6)	2 (1.8)	0 (0.0)		2 (1.9)	1 (2.8)	1 (1.6)		
5 complication	1 (0.3)	0 (0.0)	1 (0.5)		0 (0.0)	0 (0.0)	0 (0.0)		
SSI									
Superficial	25 (8.0)	8 (7.3)	16 (8.3)	0.925	8 (7.8)	4 (11.1)	4 (6.3)	0.463	1.000
Deep	0 (0.0)	0 (0.0)	0 (0.0)	–	0 (0.0)	0 (0.0)	0 (0.0)	–	–
Open wound infection	16 (5.1)	5 (4.5)	9 (4.7)	1.000	7 (6.8)	4 (11.1)	2 (3.2)	0.183	0.684
Pneumonia	10 (3.2)	3 (2.7)	6 (3.1)	1.000	3 (2.9)	2 (5.6)	1 (1.6)	0.562	1.000
Intubation	7 (2.2)	4 (3.6)	3 (1.6)	0.418	2 (1.9)	2 (5.6)	0 (0.0)	0.123	1.000
PTE	0 (0.0)	0 (0.0)	0 (0.0)	–	1 (1.0)	1 (2.8)	0 (0.0)	0.364	0.231
Renal insufficiency	3 (1.0)	0 (0.0)	3 (1.6)	0.307	3 (2.9)	2 (5.6)	0 (0.0)	0.130	0.174
Renal failure	1 (0.3)	0 (0.0)	0 (0.0)	–	0 (0.0)	0 (0.0)	0 (0.0)	–	1.000
UTI	17 (5.4)	8 (7.3)	9 (4.7)	0.490	3 (2.9)	0 (0.0)	3 (4.8)	0.307	0.422
MI	0 (0.0)	0 (0.0)	0 (0.0)	–	1 (1.0)	0 (0.0)	1 (1.6)	1.000	0.240
Bleeding	0 (0.0)	0 (0.0)	0 (0.0)	–	2 (1.9)	1 (2.8)	1 (1.6)	1.000	0.058
DVT	4 (1.3)	2 (1.8)	2 (1.0)	0.597	0 (0.0)	0 (0.0)	0 (0.0)	–	0.580
Sepsis	7 (2.2)	2 (1.8)	5 (2.6)	0.728	4 (3.9)	2 (5.6)	1 (1.6)	0.549	0.486
Return to OR	20 (6.4)	6 (5.5)	13 (6.7)	0.845	5 (4.9)	2 (5.6)	2 (3.2)	0.630	0.654
Cardiac arrest	0 (0.0)	0 (0.0)	0 (0.0)	–	0 (0.0)	0 (0.0)	0 (0.0)	–	–
CVA	0 (0.0)	0 (0.0)	0 (0.0)	–	0 (0.0)	0 (0.0)	0 (0.0)	–	–

All data represented as n (column %) unless otherwise specified

“Overall” includes non-black, non-white patients and those whose race is unknown

CVA, cerebrovascular accident; DVT, deep vein thrombosis; MI, myocardial infarction; OR, operating room; PTE, pulmonary thromboembolism; SSI, surgical site infection; UTI, urinary tract infection

### Protocol adherence

Overall, 87.5% of ERP patients (86) received or adhered to at least 12 of the 17 components for an average ERP adherence rate of 81.4%. There was no significant difference in overall adherence between black and white patients (82.3% vs 80.9%; p = 0.56) and there were no significant differences in adherence between races in any of the 17 intervention components (results not shown). Preoperative education, deep venous thrombosis (DVT) prophylaxis and antimicrobial prophylaxis interventions were achieved in every patient. Additionally, every patient was maintained at normal intraoperative body temperatures (> 36.8 °C). Multimodal analgesia administration guidelines were followed in over 97% of ERP patients. Early regular diet was initiated more than 89% of the time. Compliance with urinary catheter removal by POD 1 reached 77.3% and while not an ERP guideline, only 15 patients (15.6%) required urinary catheter reinsertion due to retention or resuscitation. 57.3% of patients achieved the guidelines for perioperative fluid management, and early mobilization at POD 1 was achieved in 37.9% of patients (Additional file 1: Table S1).

### Power

Based on prospective power calculations, for comparing the pre-ERP vs. the ERP group using two-sided t-tests and Chi-square tests with alpha < 0.05 our sample provided 80% power to detect a difference in means of 0.32 standard deviations and a difference in rates of 15% vs. 5% or 25% vs. 12%. When comparing black and white patients within the pre-ERP cohort, there is 80% power for a difference in means of 0.36 standard deviations; for comparing black and white patients within the ERP group, there was 80% power for a difference in means of 0.59 standard deviations and rates of 15% vs. 1% and 25% vs. 5%.

## Discussion

Little research has been done to assess the effect of ERP implementation within the Veterans Affairs healthcare system. In our study, we identified that ERP implementation significantly reduced pLOS for patients undergoing elective colorectal surgery. The reduction in pLOS after ERP implementation was achieved without significant increases in post-operative readmission rate or major post-operative complications. ERP patients in our study experienced an overall decreased median pLOS by 2 days, which is similar to reports of ERP effectiveness at other institutions [16, 22, 23]. In terms of secondary outcomes following ERP implementation, our complication rates are similar to those found in other studies. The rate of having any complication was 25% in our study, comparable to other reports of 22.4% [16] and 31.8% [11] and our readmission rate post-ERP implementation (16.5%) was also similar to other reports (17.6%) [16]. Higher levels of ERP adherence have been shown to improve post-operative outcomes in a dose-dependent relationship [10]. In our study, 87.5% of patients adhered to 12 or more of the 17 ERP protocol guidelines and black and white patients had similar rates of ERP compliance (85.5% vs. 88.5%, p = 0.74).

Based on findings from a similar study conducted by Wahl et al. at The University of Alabama Birmingham (UAB), a large academic medical center and our academic affiliate partner, we expected a racial disparity in pLOS to exist between black and white patients prior to ERP implementation [16]. We did find that our patient populations were racially similar (28% black vs. 35% black), however, the pre-operative lengths of stay at UAB varied by race: (black patients had an average pLOS of 10.07 days, white patients average pLOS of 7.13 days (p = 0.03) [16]) while they did not in our patients: (average 8.4 days between black patients and white patients (p = 0.67)).

Studies investigating racial disparities within the VA healthcare system are limited, though several have demonstrated significant racial disparity in clinical outcomes despite provision of medical care free-of-charge to those who qualify [24, 25]. A systemic review of racial disparities in the VA system found that while differences in outcomes exist in all clinical arenas, disparities are more frequently observed for processes associated with higher risk or which require more significant decision making by patients and/or providers (i.e. surgery/invasive procedures). Factors likely contributing to these findings include differences in health literacy, patient participation, and social support between black and white veterans [26]. While there are some studies that have shown that racial disparities within the VA Hospital System are not as apparent as in other hospital systems, our hypothesis was generated based on the findings we observed at our academic affiliate hospital [27, 28]. While we did not quantify it, our patients live in the same state, were treated under the same pathway and by the same surgeons and resident teams. Contrary to findings in non-VA settings [16], we found that racial disparities *did not exist* in patients undergoing colorectal surgery in the Birmingham VA Medical Center, though further study is needed to assess underlying factors contributing to this finding. We hope that this will stimulate future research efforts aimed at identifying the processes of care unique to the VA driving this finding.

While ERP has been shown to improve post-surgical outcomes in various hospital settings, little is known of the effects of ERP implementation within the Veterans Affairs healthcare system. Implementation of the ERP significantly reduced pLOS for both black and white patients in the VA hospital system without increasing complications, suggesting that interventions that improve the consistency of care by standardizing protocols may reduce variations in observed outcomes based on race. Lau and colleagues demonstrated this concept in addressing racial disparities in venous thromboembolism prophylaxis on a trauma service in which black and white patients received standard care 56.6% and 70.1% of the time, respectively [29]. Following standardization of venous thromboembolism prophylaxis choices, racial disparities were eliminated [29]. Like ERP, this pathway followed evidence-based algorithms to guide physicians in clinical management demonstrating that when care was applied to all patients in the same manner, the results were the same for all patients [14]. Other proposed mechanisms by which ERP programs improve outcomes for all patients include increased patient education, systematic implementation of best practices in the perioperative setting, mitigation of implicit or explicit bias, and increased adherence to evidence-based care practices [14].

Based on these findings, future work should include expanding the cases performed under ERP at our institution as well as encouraging other Veteran’s Affairs hospital to adopt ERP for their patients.

There are certain limitations of this study that are worth noting, and, as with any retrospective analysis, its findings must be interpreted within the context of the data. First, because healthcare documentation is variable amongst providers, it was difficult to acquire complete data to assess protocol adherence, although our compliance rates are high and any missing information was assumed to be nonadherent, therefore biasing our statistical decision towards the null. While our sample sizes do reflect several years-worth of elective colorectal procedures at our institution, the sample size of black patients in our intervention group (ERP) is modest (36 patients) therefore reducing the statistical power of our study. Additionally, our ERP cohort is considerably smaller than our pre-ERP cohort (103 vs. 314). Another limitation of our study is that we analyzed patient race in an isolated manner without taking into consideration other patient factors, like socioeconomic status, rurality, patient education level, or marital status, for example, that are likely also contributing to our study findings. We need to further understand and separate specific patient sociodemographic factors to understand which ones are related to surgical outcomes and which ones we can intervene upon. Lastly, our pre-ERP cohort includes patients who underwent procedures between 2010 and 2016 whereas our ERP cohort only captures patients since ERP implementation (2016–2018). The longer time period for pre-ERP inclusion may have inadvertently introduced some temporal effects.

Despite the stated limitations, our study has notable strengths compared to other studies on ERP implementation. First, we had already successfully established an ERP at a large academic medical center (our academic affiliate) in the same city. This helped decrease variation in our protocol and established guidelines for implementation in our hospital system. As a result, we modeled our study design on the ERP implementation study at that institution to help guide our assessment of outcomes. Lastly, while ERP implementation has been assessed in academic and community hospitals, evaluation within the VA Hospital System is novel.

## Conclusion

In our study, we found that there were no racial disparities in pLOS among black and white patients at our institution, and that ERP implementation significantly decreased pLOS. ERP targets the whole continuum of perioperative care and therefore can be employed to achieve health equity for surgical patients across healthcare settings and should be widely adopted.

## Supplementary Information

**Additional file 1: Table S1.** ERP Adherence Rates.

## Data Availability

The datasets used and/or analyzed during the current study are available from the corresponding author on reasonable request.

## References

[1] Haider AH, Dankwa-Mullan I, Maragh-Bass AC (2016). Setting a National Agenda for surgical disparities research recommendations from the National Institutes of Health and American College of Surgeons summit. JAMA Surg.

[2] Ravi P, Sood A, Schmid M (2015). Racial/ethnic disparities in perioperative outcomes of major procedures: results from the National Surgical Quality Improvement Program. Ann Surg.

[3] Williams DR (2012). Miles to go before we sleep: racial inequities in health. J Health Soc Behav.

[4] Schneider EB, Haider AH, Hyder O, Efron JE, Lidor AO, Pawlik TM (2013). Assessing short- and long-term outcomes among black vs white Medicare patients undergoing resection of colorectal cancer. Am J Surg.

[5] Schneider EB, Haider A, Sheer AJ (2011). Differential association of race with treatment and outcomes in Medicare patients undergoing diverticulitis surgery. Arch Surg (Chicago, Ill:1960).

[6] Ljungqvist O, Scott M, Fearon KC (2017). Enhanced recovery after surgery: a review. JAMA Surg.

[7] Girotti ME, Shih T, Revels S, Dimick JB (2014). Racial disparities in readmissions and site of care for major surgery. J Am Coll Surg.

[8] Sukumar S, Ravi P, Sood A (2015). Racial disparities in operative outcomes after major cancer surgery in the United States. World J Surg.

[9] PAR-16-392. National Institutes of Health; 2019.

[10] Gustafsson UO, Hausel J, Thorell A, Ljungqvist O, Soop M, Nygren J (2011). Adherence to the enhanced recovery after surgery protocol and outcomes after colorectal cancer surgery. Arch Surg (Chicago, Ill: 1960).

[11] Lovely JK, Maxson PM, Jacob AK (2012). Case-matched series of enhanced versus standard recovery pathway in minimally invasive colorectal surgery. Br J Surg.

[12] Grant MC, Yang D, Wu CL, Makary MA, Wick EC (2017). Impact of enhanced recovery after surgery and fast track surgery pathways on healthcare-associated infections: results from a systematic review and meta-analysis. Ann Surg.

[13] Kehlet H (2008). Fast-track colorectal surgery. Lancet (Lond, Engl).

[14] Marques IC, Wahl TS, Chu DI (2018). Enhanced recovery after surgery and surgical disparities. Surg Clin N Am.

[15] Leeds L, Alimi Y, Hobson DR (2017). Racial and socioeconomic differences manifest in process measure adherence for enhanced recovery after surgery pathway. Dis Colon Rectum.

[16] Wahl TS, Goss LE, Morris MS (2017). Enhanced Recovery After Surgery (ERAS) eliminates racial disparities in postoperative length of stay after colorectal surgery. Ann Surg.

[17] Goss LE, Morris MS, Richman JS (2018). Achieving health equity in surgery through enhanced recovery after surgery (ERAS): the elimination of racial disparities in post-operative length-of-stay is sustained long-term. Clin Nutr ESPEN.

[18] Robinson CN, Balentine CJ, Marshall CL (2010). Ethnic disparities are reduced in VA colon cancer patients. Am J Surg.

[19] Veterans Affairs Surgical Quality Improvement Program (VASQIP). Updated 2 July 2019. Accessed 7 Feb 2020.

[20] Adery CAH (1968). A simplified Monte Carlo significance test procedure. J R Stat Soc Ser B Methodol.

[21] Gustafsson UO, Scott MJ, Schwenk W (2012). Guidelines for perioperative care in elective colonic surgery: Enhanced Recovery After Surgery (ERAS(R)) Society recommendations. Clin Nutr (Edinburgh, Scotland).

[22] Geltzeiler CB, Rotramel A, Wilson C, Deng L, Whiteford MH, Frankhouse J (2014). Prospective study of colorectal enhanced recovery after surgery in a community hospital. JAMA Surg.

[23] Miller TE, Thacker JK, White WD (2014). Reduced length of hospital stay in colorectal surgery after implementation of an enhanced recovery protocol. Anesth Analg.

[24] Trivedi AN, Grebla RC, Wright SM, Washington DL (2011). Despite improved quality of care in the Veterans Affairs health system, racial disparity persists for important clinical outcomes. Health Affairs (Project Hope).

[25] Wong MS, Hoggatt KJ, Steers WN (2019). Racial/ethnic disparities in mortality across the Veterans Health Administration. Health Equity.

[26] Saha S, Freeman M, Toure J, Tippens KM, Weeks C. VA Evidence-based synthesis program reports. Racial and ethnic disparities in the VA healthcare system: a systematic review. Department of Veterans Affairs (US); 2007. 21155211

[27] Landrum MB, Keating NL, Lamont EB, Bozeman SR, Krasnow SH, Shulman L, Brown JR, Earle CC, Rabin M, McNeil BJ (2012). Survival of older patients with cancer in the Veterans Health Administration versus fee-for-service medicare. J Clin Oncol.

[28] Williams CD, Salama JK, Moghanaki D, Karas TZ, Kelley MJ (2016). Impact of race on treatment and survival among U.S. Veterans with Early-Stage Lung Cancer. J Thorac Oncol.

[29] Brandyn DL, Adil HH, Michael BS (2015). Eliminating health care disparities with mandatory clinical decision support: the venous thromboembolism (VTE) example. Med Care.

