# Supplementary material for: Patients undergoing colorectal surgery at a Veterans Affairs Hospital do not experience racial disparity in length of stay either before or after implementing an enhanced recovery pathway
Source: BMC Surg. 2022 May 21;22:201. doi: 10.1186/s12893-022-01647-3 (PMC9124421; doi:10.1186/s12893-022-01647-3)
Supplement: Supplementary file 1 — Additional file 1: Table S1. ERP Adherence Rates. [file 12893_2022_1647_MOESM1_ESM.docx]

Supplementary Table 1: ERP Adherence Rates

| **Variable** | **Overall** | **Black** | **White** | **p-value** |
| --- | --- | --- | --- | --- |
| Education | 96 (100) | 34 (100) | 62 (100) | - |
| Bowel prep | 79 (82.2) | 27 (79.4) | 52 (83.8) | 0.7746 |
| Fasting and carbohydrate treatment | 53 (55.2) | 20 (58.8) | 33 (53.2) | 0.6772 |
| Preanesthetic medication | 95 (99.0) | 34 (100) | 61 (98.4) | 1 |
| DVT prophylaxis | 96 (100) | 34 (100) | 62 (100) | - |
| Antimicrobial prophylaxis | 96 (100) | 34 (100) | 62 (100) | - |
| Standardized anesthesia | 79 (82.3) | 29 (85.3) | 50 (80.6) | 0.6012 |
| PONV prophylaxis | 91 (95.8) | 33 (97.1) | 58 (93.5) | 1 |
| Minimally invasive approach | 57 (59.4) | 19 (55.9) | 38 (61.3) | 0.6637 |
| Nasogastric intubation | 95 (99.0) | 34 (100) | 61 (98.4) | 1 |
| Hypothermia prevention | 96 (100) | 34 (100) | 62 (100) | - |
| Perioperative fluid management | 55 (57.3) | 20 (58.8) | 35 (56.5) | 0.8311 |
| Urinary drainage | 68 (77.3) | 27 (79.4) | 41 (66.1) | 0.1169 |
| Prevention of postoperative ileus | 67 (76.1) | 24 (70.6) | 43 (69.4) | 1 |
| Postoperative analgesia | 94 (97.9) | 33 (97.1) | 61 (98.4) | 1 |
| Nutrition | 79 (89.8) | 27 (79.4) | 52 (83.9) | 0.7151 |
| Early mobilization | 33 (37.93) | 13 (38.2) | 20 (32.3) | 0.6562 |

All data represented as n (column %) unless otherwise specified. For instances where the adherence rates were 100% for both black and white patients, p-values are not shown.

DVT, deep vein thrombosis; PONV, Postoperative nausea and vomiting prophylaxis.

“Overall” includes non-black, non-white patients and those whose race is unknown.
